# Metagenomic Analysis of a Concrete Bridge Reveals a Microbial Community Dominated by Halophilic Bacteria and Archaea

**DOI:** 10.1128/spectrum.05112-22

**Published:** 2023-07-05

**Authors:** E. Anders Kiledal, Mark Shaw, Shawn W. Polson, Julia A. Maresca

**Affiliations:** a Department of Biological Sciences, University of Delaware, Newark, Delaware, USA; b Sequencing and Genotyping Center, University of Delaware, Newark, Delaware, USA; c Center for Bioinformatics and Computational Biology, University of Delaware, Newark, Delaware, USA; d Department of Civil and Environmental Engineering, University of Delaware, Newark, Delaware, USA; Connecticut Agricultural Experiment Station

**Keywords:** concrete, low biomass, built environment, building materials, metagenomics, halophiles, haloarchaea, compatible solutes, ectoine

## Abstract

Concrete hosts a small but diverse microbiome that changes over time. Shotgun metagenomic sequencing would enable assessment of both the diversity and function of the microbial community in concrete, but a number of unique challenges make this difficult for concrete samples. The high concentration of divalent cations in concrete interferes with nucleic acid extraction, and the extremely low biomass in concrete means that DNA from laboratory contamination may be a large fraction of the sequence data. Here, we develop an improved method for DNA extraction from concrete, with higher yield and lower laboratory contamination. To show that this method provides DNA of sufficient quality and quantity to do shotgun metagenomic sequencing, DNA was extracted from a sample of concrete obtained from a road bridge and sequenced with an Illumina MiSeq system. This microbial community was dominated by halophilic Bacteria and Archaea, with enriched functional pathways related to osmotic stress responses. Although this was a pilot-scale effort, we demonstrate that metagenomic sequencing can be used to characterize microbial communities in concrete and that older concrete structures may host different microbes than recently poured concrete.

**IMPORTANCE** Prior work on the microbial communities of concrete focused on the surfaces of concrete structures such as sewage pipes or bridge pilings, where thick biofilms were easy to observe and sample. Because the biomass inside concrete is so low, more recent analyses of the microbial communities inside concrete used amplicon sequencing methods to describe those communities. However, to understand the activity and physiology of microbes in concrete, or to develop living infrastructure, we must develop more direct methods of community analysis. The method developed here for DNA extraction and metagenomic sequencing can be used for analysis of microbial communities inside concrete and can likely be adapted for other cementitious materials.

## INTRODUCTION

Concrete is a dry, salty, oxidized, alkaline environment that hosts a small but diverse community of microbes (1, 2). It is composed of coarse aggregate (usually gravel or crushed rock), fine aggregate (sand), cement powder, and water, with additional components depending on the performance requirements of the structure (3). Cement powder is highly oxidized and has high silica, calcium, and aluminum contents; concrete is also typically high in these elements, though this depends on the sources of the coarse and fine aggregates (4). After concrete is mixed and poured, it heats as it hardens (3). These characteristics make it a harsh, extremely low-nutrient environment.

The surface of concrete is somewhat more welcoming to microbes than the inside, since it is exposed to ambient conditions where nutrients are more available and the pH and dryness can be moderated. In fact, microbial biofilms on concrete have been well studied in sewer systems (5–11), bridge pilings (12–15), and other places (5, 16–18). These communities are abundant—often even visible to the naked eye—and reflect conditions of the surrounding environment more than their concrete substrate. However, the microbial communities inside concrete have been more difficult to study, because the high divalent cation content interferes with DNA extraction, and the low biomass complicates analysis. Despite these challenges, microbial communities in concrete have successfully been studied with DNA extraction protocols designed for concrete samples and sensitive amplicon sequencing approaches (1, 2). We have previously found that there are viable bacteria in concrete (2) and that microbial communities in recently poured concrete are influenced by the precursor materials used to produce it and local weather conditions (1).

Shotgun metagenomics is more useful than amplicon sequencing for obtaining information about microbial communities’ metabolic capabilities. However, concrete has such low biomass that metagenomic sequencing poses a real challenge: contaminating DNA from the laboratory and reagents often comprises a relatively large fraction of the extracted DNA and, therefore, a large number of the sequences obtained. We extracted DNA from road, bridge, and foundation concrete samples from Delaware and New Jersey, as well as from concrete cylinders described in reference 1 and negative control samples, to compare different extraction methods. We then used Illumina MiSeq sequencing to generate a shotgun metagenomic data set from an 81-year-old bridge in New Jersey.

Here, we report an improved method for extracting DNA from concrete samples, adapted from ancient DNA methods (19, 20), which generates DNA of sufficient quality and quantity for metagenomic sequencing. Analysis of the metagenomic data revealed that this bridge hosts a microbial community with many halophiles and haloalkaliphiles, including haloarchaea.

## RESULTS

### Improved DNA extraction from concrete.

Several modifications to our DNA extraction protocol were evaluated for their ability to increase DNA yield while limiting background contamination. We previously extracted DNA from concrete samples with a chloroform-based protocol modified from one designed for DNA extraction from ancient bone (2, 21). The first step of that protocol is a lengthy EDTA wash; longer (48 to 72 h) EDTA washes and multiple EDTA washes did not increase DNA yield (Table 1). Tests with the calcium indicator murexide showed that 10 mmol EDTA was sufficient to chelate all of the calcium leached from concrete (data not shown).

**TABLE 1:** Differences in DNA extraction yields for multiple protocols and protocol modifications^*a*^

Modification tested	Compared to	Improved yield? (*P* value)
Longer EDTA wash (10 mM, 48–72 h) followed by previous concrete protocol	Previously used protocol for concrete (1)	No (*P* = 0.32)
Two EDTA washes (10 mM) followed by previous concrete protocol	Previously used protocol for concrete	No (*P* = 0.967)
EDTA wash (10 mM, 24 h) prior to PowerMax kit	Standard PowerMax	No (*P* = 0.82)
Addition of lysozyme and proteinase K to solution C1 from PowerMax kit	Standard PowerMax	No (*P* = 0.90)
Phosphate addition (200 mM NaPO_4_) to solution C1 from PowerMax kit (22)	Standard PowerMax	No; DNA below detection limit
Qiagen low-biomass protocol	Standard PowerMax	No (*P* = 0.95)
New protocol: EDTA chelation (10 mM, 12 hours), yeast RNA carrier, guanidinium thiocyanate buffer, binding to silica suspension, and large ethanol washes	Previously used protocol for concrete	**Yes (*P* = 0.047)**

a DNA extraction yields were compared between protocols and between modified or unmodified protocols. Improved DNA yield was tested with one-sided Welch’s *t* tests; tests with a *P* value of ≤0.05 were considered a significant improvement (bold). No significant protocol modifications were identified. The new concrete DNA extraction protocol using a guanidinium thiocyanate buffer and silica suspension—scaled up from prior ancient DNA extraction techniques—significantly improved DNA yields compared to our previous chloroform-based concrete extraction protocol. The Qubit limit of detection is approximately 0.005 ng μL^−1^.

**FIG 1:**
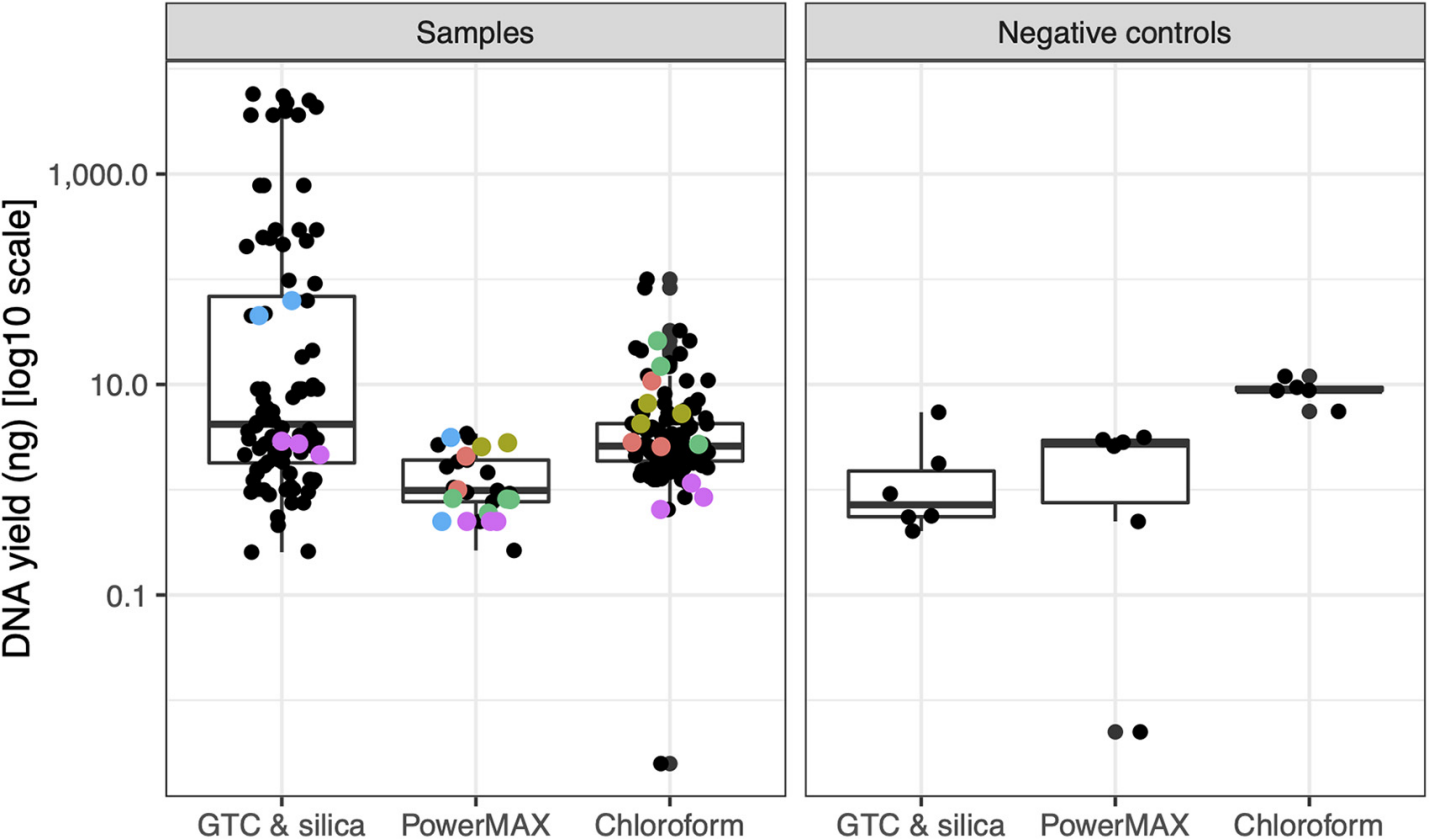
DNA yields using different protocols. Three DNA extraction methods were compared for extracting DNA from concrete: a method that binds DNA to silica particles in a guanidinium thiocyanate buffer (GTC and silica), the Qiagen PowerMAX kit, and our previous chloroform-based extraction protocol chloroform. (A and B) In each case, DNA was extracted from 10-g concrete samples; yields in ng are presented from both concrete (A) and negative-control samples (B). Colors indicate samples extracted with multiple methods. Lower negative-control extraction yields were observed with the GTC and silica protocol (one-sided Welch *t* test with PowerMAX extractions, *P* = 0.024; one-sided Welch *t* test with chloroform extractions, *P* = 0.00021).

The Qiagen PowerMax DNA extraction kit was also used to extract DNA from concrete, with and without modifications. In addition to the standard kit protocol, we tested washing the concrete with EDTA prior to lysis, adding lysozyme and proteinase K to the lysis buffer from reference 2 prior to use of the PowerMax kit, adding proteinase K to the kit lysis buffer (PowerBead solution and solution C1), adding phosphate and ethanol to the kit lysis buffer (22), and a low-biomass protocol provided by Qiagen (personal communication). These modifications did not improve yields (Table 1), and some even reduced the amount of DNA recovered. Of the methods evaluated, the PowerMax kit consistently had the smallest yields (Fig. 1), with DNA often undetectable, even using a high-sensitivity double-stranded DNA (dsDNA) Qubit fluorometric assay with the largest sample volume supported (20 μL).

Many challenges of extracting DNA from concrete are shared with extracting DNA from ancient bone and tooth samples, and the ancient DNA field more generally (19, 20, 23). These shared challenges include low-biomass samples, the related issue of contamination, and large quantities of divalent cations. A commonly used ancient DNA extraction protocol (19, 20) was adapted for concrete by scaling up the initial steps to accommodate more material while maintaining small final resuspension volumes to obtain concentrated DNA. The extraction protocol uses an initial EDTA chelation and enzymatic lysis, followed by binding of DNA to a fine silica suspension in a guanidine thiocyanate buffer. Yeast RNA is included as a blocking agent and carrier molecule. This protocol increased the DNA yield from concrete samples (Table 1), is relatively inexpensive, and requires fewer steps than either our previous chloroform-based extraction method or the Qiagen PowerMax kit. This method also decreased the yields from blank extractions (Fig. 1); in fact, only leftover primers and primer dimers were detected in our negative control extraction when assessed with an Advanced Analytical Technologies, Inc. (AATI) fragment analyzer prior to sequencing library preparation (see Fig. S1 in the supplemental material). The concentration of DNA in that sample was too low for a sequencing library to be prepared, even with a low-input DNA method.

### Shotgun metagenomic sequencing.

Illumina MiSeq sequencing generated 1,240,164 raw read pairs from the DNA extracted from concrete; no reads were obtained from the negative-control extraction. After adapter trimming and quality filtering, 1,237,961 read pairs remained. The GC content of reads was 61%. The average sequence length before trimming was 154 bp; after trimming, the average read lengths were 151 bp (forward) or 150 bp (reverse). Reads were assembled with metaSPAdes (24); however, most contigs were less than 2,000 bp, which precluded effective binning and metagenome-assembled genome (MAG) recovery.

### Community analysis.

Reads were first classified with Kraken 2 against the Genome Taxonomy Database (GTDB), successfully classifying 767,469 reads (61.99% of the total) as either Bacteria (48.8%) or Archaea (12.8%). Reads unclassified against GTDB (12,155 reads; 0.98% of the total) were further classified with Kraken 2 against a RefSeq database including eukaryotes (0.46%) and viruses (0.0036%).

**FIG 2:**
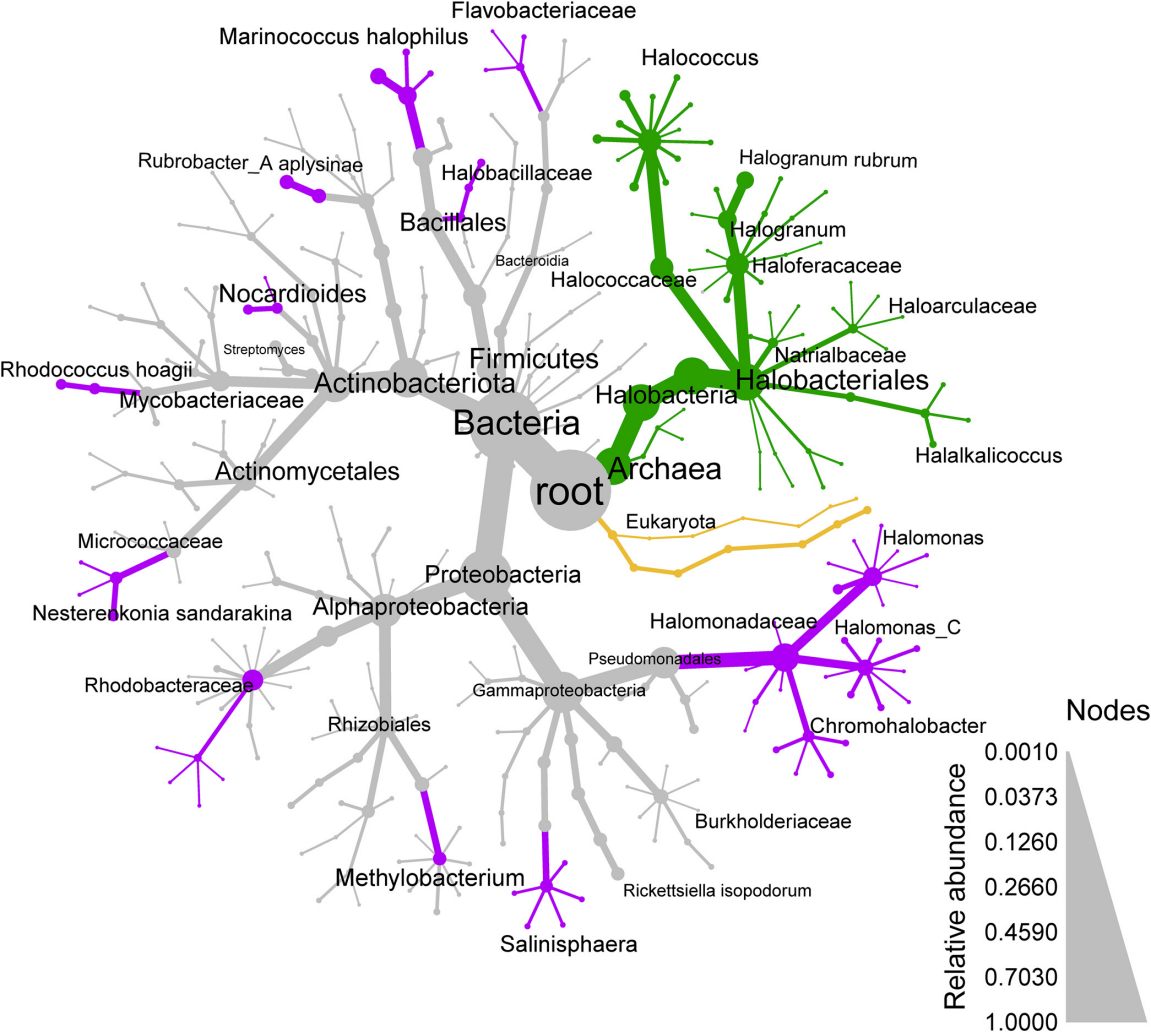
Microbial community composition in concrete. Most of the organisms observed were Bacteria (78.6%) and Archaea (20.6%), while eukaryotes and viruses were observed in much lower abundances. All of the observed Archaea are putatively halophilic organisms (green nodes and lines), while many of the observed Bacteria are also likely halophilic (purple nodes and lines). Node size and edge thickness indicate relative abundance. Only groups with relative abundances of >0.001 are shown; however, excluded taxa were included in the abundance calculations of higher-rank taxa.

Considering the relative abundance of organisms (percentages that follow are of the 767,469 classified reads), the majority of organisms observed were Bacteria (78.6%), specifically *Proteobacteria* (42%), *Actinobacteria* (24%), *Firmicutes (Bacillota)* (7.6%), and *Bacteroidota* (2%) (Fig. 2). *Gammaproteobacteria* amounted to 25% of total observations, particularly halophilic organisms in the *Halomonadaceae* family (12%), which includes *Halomonas*, the most abundant genus observed (9%), *Marinobacter* (1.3%), and *Salinisphaera* (2.3%). *Alphaproteobacteria* were also abundant (17%), particularly *Rhodobacteraceae* (6.4%), *Methylobacterium* (2.5%), *Rhizobiaceae* (2.4%), and *Sphingomonadaceae* (1.2%). The phylum *Actinobacteriota* represented 24% of the community, with most belonging to the class *Actinomycetia* (19%). In the actinobacterial class *Rubrobacteria*, the genus *Rubrobacter* (3.5%) was also abundant. *Firmicutes (Bacillota)* were 7.7% of the community, particularly the genera *Marinococcus* (4.9%) and *Halobacillus* (0.96%).

Archaea (20.6%), almost exclusively from *Halobacteria* (20.5%), were abundant in this community and included the species with the highest relative abundance, Halogranum rubrum (4.4%; Fig. 2). The genus *Halococcus* made up 7.93% of the community, and we also observed other members of the order *Halobacteriales*, such as *Natrialbaceae* (1.41%) and *Halalkalicoccus* (1.2%).

Eukaryotes (0.73%) and viruses (0.0051%) represented only small proportions of the classified reads. The most abundant eukaryotic groups included plants (Streptophyta, 0.56%) and fungi (0.045%). Most metazoan reads (0.12%) were human (0.118% by Kraken, 0.077% by mapping to the human genome), likely contamination, while fungi were mostly the plant pathogens *Colletotrichum* and Fusarium. A separate community analysis using the lowest common ancestor (LCA) of reads mapped to the UniRef100 protein databases also confirmed low fungal abundance (0.76%) (Fig. S2). The viral sequences observed were mostly bacteriophage of Mycobacterium, *Gordonia*, *Rhizobium*, or Acinetobacter.

### Functional analysis.

Enrichment of biological process gene ontology (GO) terms in the concrete metagenomic data relative to all GO annotations of the UniRef90 database was determined by mapping UniRef90 clusters to GO terms and testing for .enrichment with the topGO R package (25) (Table 2) Some of the enriched GO terms include general metabolic strategies such as carbohydrate metabolism, urea catabolism, ethanol oxidation, and photosynthesis. Enriched GO terms related to stress responses include DNA protection (26) and heavy metal detoxification, specifically responses to nickel and mercury ions (27). Additionally, several pathways related to compatible solutes are enriched, including synthesis and catabolism of ectoine (28, 29), spermidine and putrescine (30, 31). Finally, GO terms associated with *Halobacteria* were enriched, including gas vesicle formation (32). Complete results of the GO term analysis are available in File S3.

**TABLE 2:** GO terms enriched in metagenomic data^*a*^

GO ID	*P* value	GO term (biological process)
GO:0001123	9.50E-07	Transcription initiation from bacterial-type RNA polymerase promoter
GO:0052837	1.69E-05	Thiazole biosynthetic process
GO:0009767	1.67E-04	Photosynthetic electron transport chain
GO:0010045	5.08E-04	Response to nickel cation
GO:0006189	6.98E-04	*De novo* IMP biosynthetic process
GO:0009229	6.98E-04	Thiamine diphosphate biosynthetic process
GO:0006069	6.98E-04	Ethanol oxidation
GO:0070897	7.28E-04	Transcription preinitiation complex assembly
GO:0042400	9.79E-04	Ectoine catabolic process
GO:0039684	0.00117427	Rolling-circle single-stranded viral DNA replication
GO:0009758	0.001850471	Carbohydrate utilization
GO:0006614	0.002876243	Signal recognition particle (SRP)-dependent cotranslational protein targeting to membrane
GO:0046797	0.002933102	Viral procapsid maturation
GO:0050787	0.004896114	Detoxification of mercury ion
GO:0042262	0.005014208	DNA protection
GO:0036104	0.010020618	Kdo2-lipid A biosynthetic process
GO:0045905	0.012693054	Positive regulation of translational termination
GO:0070929	0.012693054	Trans-translation
GO:0101030	0.012693054	tRNA-guanine transglycosylation
GO:0019491	0.012693054	Ectoine biosynthetic process
GO:0009249	0.012693054	Protein lipoylation
GO:0031412	0.012693054	Gas vesicle organization
GO:0009097	0.012693054	Isoleucine biosynthetic process
GO:0009099	0.012693054	Valine biosynthetic process
GO:0071793	0.012693054	Bacillithiol biosynthetic process

a GO terms enriched in the metagenomic data relative to the UniRef90 cluster GO annotations were identified with the topGO R package, using the weight01 algorithm and Fisher’s exact test (null hypothesis: GO term abundance is the same in the GO term database and concrete metagenomic data). The top 25 most enriched GO terms are included in this table, along with the *P* value of the enrichment. A complete list of GO terms and the probabilities that they are enriched in this data set is available in File S3.

**FIG 3:**
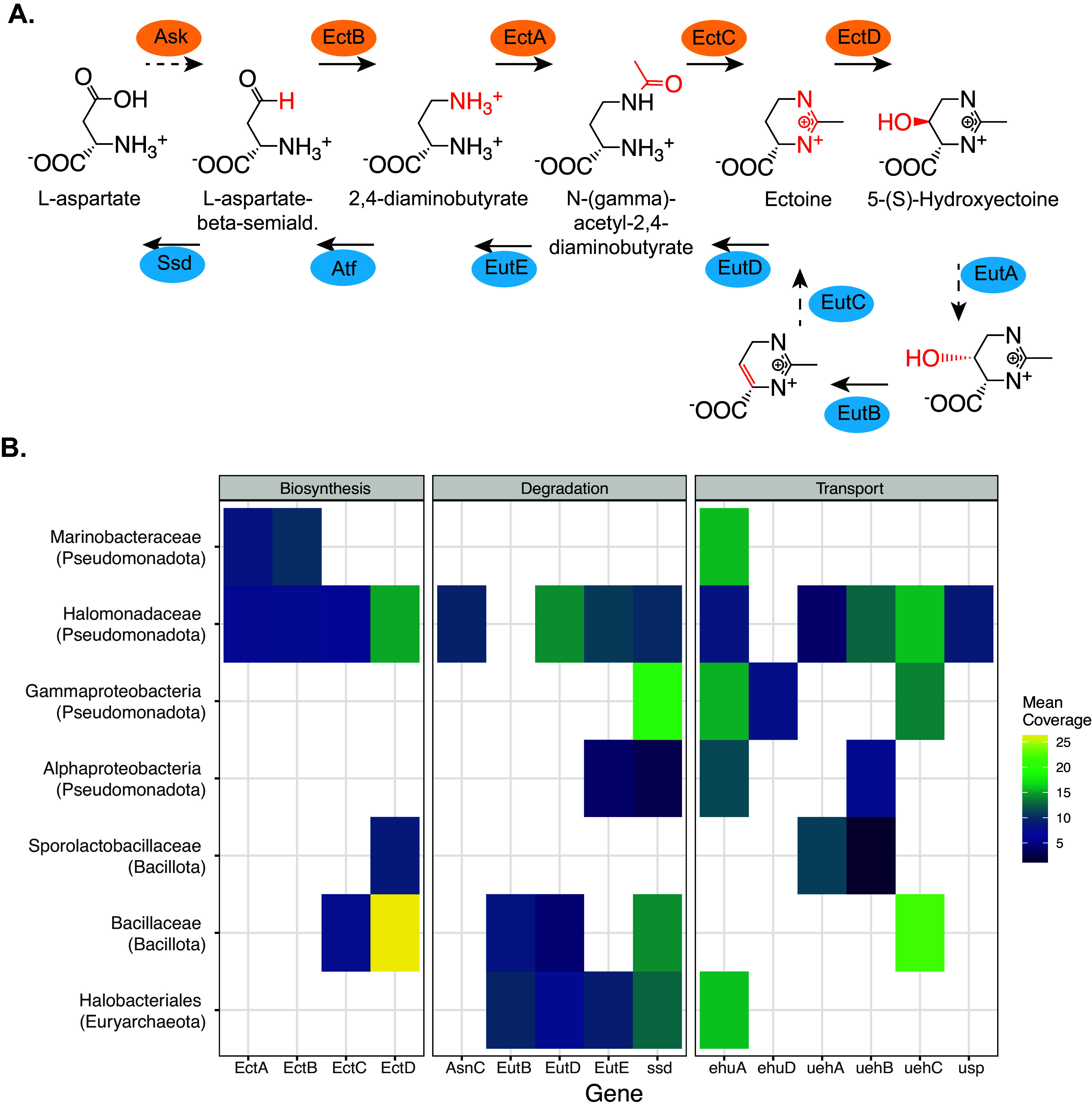
Ectoine synthesis, degradation, and transport. (A) Biosynthesis and degradation pathways for ectoine and hydroxyectoine, after Schulz et al. (29). (B) Genes related to ectoine synthesis, degradation, or transport are found in contigs from a phylogenetically diverse range of microbes, including *Halobacteria*, several classes of *Actinobacteria*, and *Proteobacteria*. Color indicates the depth of coverage for the contig with the specified genes. Genes were summarized at the family level, and only family or higher ranks with two or more observed genes are shown.

Ectoine and hydroxyectoine are compatible solutes produced by Bacteria and Archaea in high-salt environments (29, 33, 34). They are synthesized from aspartate by EctBAC(D), and ectoine is imported into cells by the UehABC transporter and degraded by EutDE-Atf-Ssd (Fig. 3A) (29).

Of the known (hydroxy)ectoine biosynthesis genes, only EctB was observed in contigs from Archaea (*Halogranum* and *Halalkalicoccus*). Homologs of EctCD (the ectoine and hydroxyectoine synthases) were observed in contigs originating from both *Bacillota* and *Actinobacteria*. The *Gammaproteobacteria* appear to encode all four (hydroxy)ectoine biosynthetic genes, the ectoine transporter, and all of the necessary genes for degradation of ectoine (Fig. 3B). The *Halobacteria* appear to encode all of the necessary genes for ectoine degradation.

Oxygen availability may vary through the concrete matrix, so the predicted oxygen usage and tolerance of community members was profiled by (i) comparison to annotations in the BacDive microbial phenotype database, (ii) computational trait prediction for reference genomes most similar to community members identified with sourmash, and (iii) the presence of key genes from anaerobic processes. Genera with BacDive annotations accounted for 51.42% of the community abundance, and the vast majority were predicted to be aerobes (49.5%), although some putative facultative anaerobes (0.5%) and obligate anaerobes (0.3%) were also identified. Genomic trait predictions were similar; Traitar predicted oxygen sensitivity traits for 10 of the 15 reference genomes. Seven were predicted to be aerobes, two were predicted to be facultative anaerobes, and one was predicted to be capnophilic. Two of the more abundant predicted obligate anaerobes were both *Bacillota*, *Halobacillus* (obligate; BacDive) and Marinococcus halophilus (facultative; Traitar). Genes involved in anaerobic processes were also observed among the top hits of reads mapped to UniRef100 gene clusters, including fermentation (lactate dehydrogenase, alcohol dehydrogenase, formate dehydrogenase), dissimilatory nitrate reduction (nitrate reductase), and denitrification (nitrous-oxide reductase) (Table S4).

Because biofilms were observed on the surfaces of some concrete pieces (Fig. S3), the potential for biofilm formation was assessed using read mapping to UniRef100 gene clusters involved in biofilm formation. Biofilm polysaccharide poly-β-1,6-*N*-acetyl-d-glucosamine synthesis *N*-glycosyltransferase *pgaC* genes from Halomonas utahensis and Pseudomonas indica were 96.8% (mean depth, 8.7) and 90.7% (mean depth, 8.2) covered by reads, respectively. Reads also mapped to a *pgaB N*-deacetylase gene from *Halomonas* (coverage, 99.2%; mean depth, 23.4). Genes involved in other biofilm-related processes were also observed from Marinococcus luteus (cell fate regulator YlbF; coverage, 100%; mean depth, 16.5), *Oxalobacteraceae* bacterium OM1 (biofilm regulation diguanylate cyclase SiaD; coverage, 63.6%; mean depth, 10.6), and *Nesterenkonia* spp. (cyclic-di-GMP-binding biofilm dispersal mediator protein; coverage, 98.6%; mean depths, 5.9) (Table S5).

## DISCUSSION

Here, we analyzed DNA extracted from an 81-year-old, alkali-silica reaction (ASR)-affected concrete bridge in New Jersey and found a microbial community dominated by halophiles, with a large number of halophilic Archaea, and very few eukaryotes. These microbes encode pathways that potentially allow them to mitigate the primary selective pressures in concrete, including heavy metal tolerance, biofilm formation, and a variety of osmotic stress responses. This contrasts with our previous work in much younger concrete (<3 years old), which used 16S amplicon sequencing to characterize a microbial community composed primarily of generalist, sometimes oligotrophic, Bacteria and no detectable Archaea (1). Although the biomass and therefore DNA concentration in concrete is extremely low, with larger sample sizes and this improved extraction method, metagenomic sequencing is feasible without prior amplification.

The microbial community in this concrete bridge is highly enriched in halophiles and haloalkaliphiles. The differences between this sample and those analyzed in our prior work suggest that the microbial community in concrete can indeed change dramatically with time and, thus, that concrete itself is a dynamic (if slow) environment. As concrete ages and reacts with CO_2_ in the atmosphere, the pH decreases to ~9 (35). Further, in structures affected by ASR like the one studied here, a hygroscopic, alkali-rich gel is formed inside the concrete and can lead to extensive cracking (36). This damage allows water to enter the structure, which likely delivers nutrients to the interior of the concrete but also solubilizes ions in the concrete matrix. Any microbes able to obtain nutrients from the water would thus also have to tolerate a solution with extremely high salt concentrations and likely high pH (37). This strong selective pressure is reflected in the microbial community, which includes not just known halophiles, but also haloalkaliphiles such as *Halalkalicoccus* and *Natrialbaceae* (38, 39).

The differences between the microbial community in this aged, damaged structure and those in young (less than 3 years old) concrete suggests that the microbial communities may change over time. The large number of reads attributable to Archaea (~30%) were a surprise, since no Archaea were detected in any of the samples in our previous work. The primers used in our previous study (357F/806R) do not amplify 16S genes from *Halobacteria*, so it is unknown whether Archaea were not observed due to primer specificity or absence/low abundance in the samples.

The current results raise the very interesting question of where the microbes in concrete might come from. Our prior work indicated that, at least initially, the components of concrete “seed” microbes in concrete. Many of these microbes die within a few months, presumably unable to withstand the conditions in concrete. It is possible that the halophiles in this New Jersey sample descended from a very small number of halophiles present in the initial concrete mix. We cannot reconstruct the microbial community of freshly poured concrete from its current microbial community; however, we saw little evidence of halophily in the microbial communities of the components of concrete. Alternatively, halophiles could have been introduced by wind, rain, road salt, or other vectors. *Halomonas* and *Halobacillus* spp. were observed in freshwater and soil microbial communities impacted by deicing salts (40–42), and the phylum *Euryarchaeota*—which includes the haloarchaea—composed ~0.2% of the microbial community detected by amplicon sequencing in a road salt storage box in the Baltimore, Maryland, area (42, 43). Additionally, most of the haloarchaeal genera observed in this concrete sample were also found in halite, including *Haloferax*, *Haloarcula*, *Halalkalicoccus*, *Halococcus*, and *Natrialba* spp. (43), suggesting that rock salt is inhabited by these species and could potentially seed similar high-salt environments with halophiles.

Also somewhat surprising was the low fungal abundance. Fungi, particularly lichens, can colonize concrete surfaces (44), and a few have been isolated from concrete (45). It is possible that some of the nearly 40% of reads that could not be classified belong to poorly characterized fungal groups, since databases of fungal genomes lag significantly behind those for Bacteria and Archaea (46). However, low fungal abundance was consistently observed across multiple bioinformatic approaches using different reference databases.

This work demonstrates that with an improved, scaled-up method for DNA extraction, shotgun metagenomic analysis of microbial communities within concrete is feasible. This method will facilitate analysis of concrete microbiomes in studies of environmental factors that contribute to infrastructure support or degradation, bio-repair of structures by endogenous, bio-augmented, or designed microbial communities, or in efforts to engineer living materials. Here, we show that the microbial community in aging structural concrete is dominated by putatively aerobic, halophilic species and that the microbial community in old concrete is quite different from microbial communities in relatively new concrete. Perhaps if microbes are added to concrete mixes for future bio-repair, a cocktail of strains that will persist and perform at different stages of the concrete service lifetime will be necessary.

## MATERIALS AND METHODS

### Sample collection and processing.

The concrete sample that was sequenced was collected from the U.S. 206 bridge over Dry Brook in Sussex County, New Jersey (GPS coordinates 41.140338, −74.743574). To avoid damaging the structure, the sample was collected from a large piece of concrete that had broken off from the underside of the bridge deck. A roughly 500-cm^3^ sample was wrapped in multiple layers of aluminum foil, transported at ambient temperature, and frozen at −80°C the same day. Prior to DNA extraction, concrete fragments were ground to powder in a ring and puck mill that had been scrubbed with soap and hot water, dried, and then cleaned twice with ethanol and UV-irradiated for 40 min. A negative-control extraction blank was also prepared using 10 g of triple-sterilized glass beads.

### DNA extraction.

To increase the DNA yield and decrease contamination introduced during DNA extraction, we tested a number of modifications to the protocol (see File S1). After development of the modified protocol, DNA was extracted from 10 g of ground concrete from a New Jersey bridge and a blank negative control extraction. After grinding, all materials were handled inside a UV- and bleach-decontaminated PCR hood. DNA was quantified using an Invitrogen Qubit fluorometer (version 2) with a Qubit dsDNA high-sensitivity (HS) assay kit (Thermo Fisher, catalog no. Q32851).

Samples were incubated overnight with gentle rotation at 55°C with 5 mL 0.5M EDTA, 150 μL proteinase K (800 units mL^−1^; New England Biolabs, no. P8107S), 138 μL 20% SDS, and 0.2 mL acetic acid in a 50-mL conical tube, scaled up from methods previously described (19, 20, 47). After incubation, samples were vortexed at maximum speed for 10 min on a multitube vortexer (Dade, catalog [cat.] no. S8215-1). Samples were then centrifuged for 3 min at 5,000 rpm, and the supernatant was transferred to a new 50-mL conical tube. Then, 30 mL of modified QG buffer (5 M guanidium thiocyanate, 18.1 mM Tris-HCl, 25 mM NaCl, 1.3% Triton X-100) was added to the supernatant with 5 μL yeast RNA (1 mg mL^−1^; Sigma-Aldrich, no. R6625) and 125 μL of a silica suspension prepared as previously described (47). DNA was allowed to bind overnight to silica particles at room temperature with gentle mixing (~30 rpm). Following binding, samples were centrifuged for 5 min at 5,000 rpm, and the supernatant was discarded. The silica pellet with bound DNA was washed by resuspension in 10 mL 80% ethanol and centrifuged for 10 min at 5,000 rpm. The supernatant was again discarded, and the pellet was resuspended in 1 mL 80% ethanol, transferred to a 1.5-mL microcentrifuge tube, and centrifuged for 3 min at 13,000 rpm; the supernatant was discarded. The silica pellet was air dried in a laminar flow hood. DNA was eluted from the silica twice in 50 μL prewarmed (60°C) 10 mM Tris for 5 min with gentle rotation at 60°C. For each elution, the silica was pelleted for 1 min at 13,000 rpm prior to recovery of eluted DNA. This protocol has been published online with additional comments (48).

### Library preparation and sequencing.

Prior to sequencing and library preparation, DNA was quantified with an AATI Femto Pulse. Sequencing libraries for the bridge sample and a negative control sample were prepared with the Illumina Nextera XT kit following the manufacturer’s instructions with three modifications: half reactions due to low input DNA, 12.5 min tagmentation, and 20 PCR cycles. Then, 1 ng of the bridge sample DNA and 5 μL of the negative control sample were used as starting material. Library quality and sizing were analyzed with an AATI fragment analyzer. The initial bridge sample had a library size distribution of ~150 bp to 1,600 bp and was subsequently size-selected using a Sage Scientific BluePippin instrument, resulting in a final library with ~350- to 650-bp fragments. The negative-control library preparation contained only leftover primers and primer dimers. The bridge sample library was sequenced on an Illumina MiSeq device with 150-bp paired-end reads.

### Bioinformatic and statistical analyses.

**(i) Quality control.** Reads were filtered and trimmed with TrimGalore version 6.6 (49). FastQC version 11.9 (50) was used to generate quality reports, which were summarized using MultiQC version 1.6 (51). Human reads were identified by mapping to the human reference genome GRCh38 with the Minimap2 aligner version 2.24-r1122 (52) via CoverM version 0.6.1 (53) (parameters: –contig-end-exclusion 0 –min-covered-fraction 0, -m relative_abundance mean covered_bases variance length count rpkm tpm). Bioinformatics, data processing, and statistical analyses were conducted with a custom snakemake workflow available on GitHub (https://github.com/MarescaLab/concrete_metagenome_test). Software dependencies in the workflow ere managed by Conda and Docker to aid reproducibility.

**(ii) Taxonomic analysis.** The k-mer-based tool Kraken 2 version 2.1.1 (54) was used to generate a taxonomic profile from paired, quality-controlled reads. Reads from Bacteria and Archaea were first classified against a Genome Taxonomy Database (GTDB) release 95 database (55) produced with Struo (56) and available at ftp.tue.mpg.de/ebio/projects/struo/GTDB_release95/. Reads from Eukarya and viruses were classified against a precomputed RefSeq database (PlusPFP 1/27/2021) available at https://benlangmead.github.io/aws-indexes/k2. From Kraken 2 taxonomic profiles, Bracken (57) was used to calculate species-level relative abundance with otherwise default parameters. Community composition was also assessed by mapping reads to the UniRef100 protein reference database and computing the lowest common ancestors of hits with the easy-taxonomy workflow in mmseqs2 version 14.7e284 (parameters: –lca-mode 3 –orf-filter 1 –orf-filter-s 3.5 –db-load-mode 2 -s 4). The minimal set of reference genomes covering the metagenome was determined using sourmash (58, 59) and a GTDB database, and the identified references were downloaded for subsequent analysis.

**(iii) Assembly.** Reads were assembled with metaSPAdes (24) version 3.14.1 using default parameters.

**(iv) Functional analysis.** General functional annotation was performed with the HUMAnN3 tool (60), using a GTDB release 95 (55) database produced with Struo2 (56, 61) (available at http://ftp.tue.mpg.de/ebio/projects/struo2/) and taxonomic annotations produced with Kraken 2 and Bracken (54, 57). The abundance of UniRef90 gene clusters (62, 63) was determined using the HUMAnN3 tool (60), and a mapping file provided by the authors of the HUMAnN3 tool was used to map Gene Ontology (GO) terms to the UniRef90 clusters. Genes involved in biofilm formation and anaerobic processes were identified from read mapping top hits to UniRef100 gene clusters with mmseqs as described in the taxonomic analysis methods above.

Queries of BacDive (64) for organisms with oxygen tolerance annotations were conducted for the 1,000 most abundant genera in the metagenome using R scripts (available at github.com/MarescaLab/concrete_metagenome_test). Additionally, traits including oxygen tolerance were predicted using a python3 implementation of Traitar (65, 66) for the reference genomes identified above with sourmash.

Sequences of bacterial and archaeal proteins involved in ectoine biosynthesis, transport, and degradation (28, 29) (see File S2) were used as queries in a TBLASTN (version 2.11.0 [67]) analysis to identify corresponding genes in the metaSPAdes assembly. Hits were retained if they satisfied the following filtering criteria: E-value, <0.001; Bit_Score, >60; Percent_ID, >40; percent_of_query_aligned, >20. Contig taxonomy was determined with Kraken 2 version 2.1.1 (54) and a Genome Taxonomy Database (GTDB) release 95 database (55) produced with Struo (56) and available from ftp.tue.mpg.de/ebio/projects/struo/GTDB_release95/. Mean contig coverage was determined by mapping reads to contigs with CoverM version 0.6.1 (53) and the Minimap2 aligner (52).

**(v) Statistical analysis.** Enrichment of biological process GO terms was calculated with the topGO R package, using the default weight01 method, which assigns greater weight to more specific GO terms (25, 68).

### Data availability.

Raw and assembled DNA sequences are available at the NCBI, associated with BioProject PRJNA846790.
