# Supplementary material for: Metagenomic Analysis of a Concrete Bridge Reveals a Microbial Community Dominated by Halophilic Bacteria and Archaea
Source: Microbiol Spectr. 2023 Jul 5;11(4):e05112-22. doi: 10.1128/spectrum.05112-22 (PMC10434110; doi:10.1128/spectrum.05112-22)
Supplement: Supplemental file 2 — File S2. Download spectrum.05112-22-s0005.pdf, PDF file, 0.02 MB [file spectrum.05112-22-s0005.pdf]

> WP_007424000.1 ; ACRY_RS17550 ; Acry_3007 ; MarR family transcriptional regulator ; EctR
MDEDLTSATLRAIRRILRATDQGSHRLYAATGLTTSQFLVLQEIDRRGDATPGTIATALQFGQATITNIA
DRLVAAGLITRRRGERDKRQVILAATDAGRAALGRAPDLLQERFRDRFQGLPLWERAMILAALERVGSLL
DASGIDAAPLIAAGAIDRAAGPPAEAEPAPRQDAAPAIGHPS

>WP_012040477.1 ; ACRY_RS17555 ; Acry_3008 ; diaminobutyrate acetyltransferase ; EctA
MALRRPTAADGPAVTALIADCPPLDANSAYCNLLQCTDFAETCVLAEREGAVVGWISGYRPPSDLSRIFV
WQVAVSSAARGIGLGGQMLDALLDRPAIAGVRALTTTITEANTASWRLFESLARRRGGSFARAVRFDRAA
HFAGLHDTEFEVTIGLPQPVAEPARKDIS

>WP_012040478.1 ; ACRY_RS17560 ; Acry_3009 ; diaminobutyrate--2-oxoglutarate transaminase ; EctB
MTDLPRPSRRKPDTAIYERMESRVRSYCTAIPRQFTRAEGAWLHDSHGGRYLDFLAGCSSLNYGHNHPVL
KRVLIDYIARDGIAHGLDFHTDAKAEFLDALETVILKPRGMDYRAMFVGPTGTNAVEAALKLARKVTGRQ
TVIAFTNGFHGMTLGALACTGNEGKRGGAGVPLAHVSHEPYDGYYGPEIDTADLLEQRLADGSSGLDAPA
AFLVETVQGEGGLNAASAGWMRKIAALAKRHGALLIVDDIQAGCGRTGRFFSFEEMGIRPDIITLAKSLS
GIGAPFALTLFRPELDQWKPGEHNGTFRGNNHAFVTAAAAIRHFWSDDAFAADIARRGDLLGRRLDAIAA
RHGMGTRGRGMMQGIDAGTGEMAERITGIAFREGLVIETSGAGGEIVKVLAPLTIDDDLLAAGLDILERA
VNEATAPTYSVAAE

>WP_012040479.1 ; ACRY_RS17565 ; Acry_3010 ; ectoine synthase ; EctC
MIIRTLKEAQASERKVVTDGWDSVRMLLRDDGMGFSFHITTMYAGKELRMHYKHHYEAVFVLSGEGMIED
LDAGETHQLKPGVLYVLNKHDRHVVRPKTDIVTACVFNPPVTGQEVHDASGAYPPAPVGEAA

>WP_012040480.1 ; ACRY_RS17570 ; Acry_3011 ; ectoine hydroxylase ; EctD
MDDLYPSRREPTPSLLPRHDPVVHGRWAPGAPLSDEQTRFYDTNGYLVLENVFDPAEIALLQSGSMDLLA
NPAGLDRETIITERGSDEVRSIFAIHAQNELLGRLAADSRIAGVARFLLDDDVYIHQSRLNYKPGFDGKE
FYWHSDFETWHVEDGMPRMRALSMSILLAENTANNGPLMVIPGSHRKYLTCVGETPENHYRSSLKKQEYG
VPDREMLTALASDHGIVAPTGKAGTVVLFDCNTMHGSNGNITPFPRSNAFFVFNAKANSLVEPFGPKSRR
PDFIADRAFTTVDIVKGPLVRRERAA

>WP_012040481.1 ; ACRY_RS17575 ; Acry_3012 ; aspartate kinase ; ask_ect
MSMAPSVEKIGGTSIAATDAVVGNVLIAGRAGRDLYRRIFVVSAYGGITDLLLEPKKKTDAAKPPGLYAS
FAADGEKGDWRDALDAVAAAMRARNEEVFGTSPERAVADDFVAARIGETRACLDDLDRLRGHGHFRLDEP
LATLRELLAGLGEAHSAHNTALLLRARGVNAAFVDLTGWQDGRNLDLDERIRTGLDGIDSATTLPIVTGY
VKCSDGMVRKYDRGYTEMTFARLAVLTGAREAIIHKEFHLSSADPKVVGVGKARKIGRTNYDVADQLANL
GVEVIHPGAARGLRQAGIPLRVRNTFDRHDEGTLICADYVSAAPRIEIVTGIRELRALQFFEQDMVGKKG
YDAAILDTLTKHRARIVSKSSNANAITHYLAAGGPTVRRVIADLEARFPNAEVSAPRLAMVALIGSDLSA
EGCVGRALDALGAAGIGVKAMQHQMRNVDIQFILDTDRFDDAIRALHAALVECRATQDGKGGTLRTAA

>WP_011046895.1 PLP-dependent aminotransferase family protein ; enuR [Ruegeria pomeroyi]
MTNWLPDPSALASPLHSSLALAIAKAISEGKLPKGTQLPTHRKLAEELGLSVHTVSKAYESLRRQRLIDG
QVGRGSYVLDLNTPDDQPFQLSSERGRNFDLSISRPAFSQRHAELFQQVLADLPQGLDPSYYLACRPNVG
HDAHRAAGETWLRICGLDVPSERIIMTNGVSHGMSAALSALARPGDTVVSSRITHHLLVSGCSYLGLNLV
GIDFDQDGMLPDALDRHCSENNAKVLFLLPSLANTTVEMIPEERRQALVQVARKHNLYIIENDAFGPVAE

DRPVPVAALAPERTIYLTTFTKCTVSGLRAGYMAAPEHLLPALTGRIVVFGWMATPLMCEIATRWVLDGT
ALELALWQRRALAERYEIASQALQGHQWRGHPSALHLWLPLESGWTTSSFVAYARQLKVAVAPDAPFLTP
KTPPPNAVRISLGSVQDLSRFKQAMELVGGMLSRPPEGVAPLAF

>WP_011046894.1 TRAP transporter substrate-binding protein DctP ; uehA [Ruegeria pomeroyi]
MAQSITFTFGAVAAAGIALAAGTAAQADTWRYAFEEAMTDVQGVYAQKFKEEIEANSDHEIQLFPYGTLG
ESADIMEQTQDGILQFVDQSPGFTGSLIPEAQVFFVPYLLPTDQDHLARFFKESKAINDMFKPLYADQGL
ELLNMFPEGEVAMTTKTPVTTCSDLDEVKFRVMTNPLLVESYKAFGATPTPLPWGEVYGGLQTNVIQGQE
NPTFFLYSTKIYEVTDYITYAGHNNFTTAVMANKDFYDGLSAEDQQLVQNAALAAYDHTVVYQQQAADTE
LAKIMEAKPEMQVTVLTDEQRSCFKEAAAEVEAKFIEMTGDSGAAILKQMKADLAATAN

>WP_011046893.1 TRAP transporter small permease ; uehB [Ruegeria pomeroyi]
MQQTHESALPGFLGMLDSAISRIESFLLAMGVLLMAANTVANVVGRFVLGNSIFFSEELNRILIILITFA
GISYAARNARHIRMSAVYDLLPARLRKGMMVVISVVTAAFMFLLCYYAAKYIGSQASRGRVLPALQIPVW
VILIWVPAGFFMTGAQYLLTAVRNLTSSGIYLSTHVQEGYEDAEEIEI

>WP_011046892.1 TRAP transporter large permease ; uehC [Ruegeria pomeroyi]
MAATIFLTMIVLLLLGFPMMIPLIAGAFIGFLMLFGDLARTETMVQQMLAGIRPASLIAVPMFIFAADIM
TRGQSAGRLINVVMAYVGHIRGGLAISTAAACTMFGAVSGSTQATVVAIGSPLRPRMLKAGYKDSFVLAL
IVNASDIAFLIPPSIGMIIYGVVSSTSIAELFIAGIGPGLLILVLFSAYAYIYAVRNDVPTEPRASWAER
ARTMRQALWPMGFPVIIIGGIYGGVFSPTEAAAACVLYALVLEVLVFRSMSLADVYDTAKSTGLITAIVF
ILVGAGAAFSWVISFAQVPQQILGAIGIAEMGPIGVLFVISIAFFIGCMFVDPIVVILVLVPVFAPVVKS
VGLDPVLVGTIITLQVAIGSATPPFGCDIFTAIAVFKRPYAEVVRGTPPFILMLLGVSVALIFFPQIALF
LRDLAFSK

>WP_011046891.1 universal stress protein ; usp [Ruegeria pomeroyi]
MFASILVPFDGSHGAEAALAKAAALACLCGAELTLLTVVYRHHSPLEASMHVVRPDEPADLDDIMRTHARE
VAEHGKARAAAYGVPAPRAFVKGGPVARTIAGFAKEHGHDLTVIGSRGLGSFERALLGSVSHKVTSLSET
PVLVV

>WP_011046890.1 ectoine utilization protein ; EutA [Ruegeria pomeroyi]
MALDTHSAPSRVPARLDDRPVRKRIALVALATDHTSERDFARICDPDQVGVYTNRIAFENPTNKETLLKT
GPRLTEAAARILPGEEVDVVAYCCTAASIVLGNDTVTRHLNAAKPGTPCVTPSSAAFDAFAALGVRRVSL
LTPYSPDVTDEMAQYFAVHGPEVVNAACFGLTDDREMARISEDCIIEAALAAFDKDAEALFLSCTALRAA
TCAQRIEDRLGKPVVTSNQAMIWRCLRLAGIPDMVPGYGRLFHL

>WP_011046889.1 hydroxyectoine utilization dehydratase ; EutB [Ruegeria pomeroyi]
MTTKITLQDSIRARARIAGRIRETPLVESPSLSSLSGRQVLLKLESRQITGSFKLRGATNAVLSLTDTQR
AAGVVGVSTGNHGRGLAYAAAEAEVRCIICMSELVPQNKVDGIRSHGAEVRILGRSQDDAQQEVDRLVRD
GMTMLPPFDHPDIIAGQGTVALEMLDQAPDLETVLVPLSGGGLISGVGMVLKAANPDIRVIGVSMERGAA
MYECLQAGRPVQVAELPTLADSLGGGIGLDNAYTFEMTKAFVDEVVLVSEAEIAAAIRHAYFEEREVIEG
SGSVGIAALLAGKIGNPGRCVSLVSGQNIAMDLHKRIIDGEDVDVEADIKGGADA

>WP_044028000.1 ectoine utilization protein ; EutC [Ruegeria pomeroyi]
MPDIRILTEAELRDLVPLDLDAVDCVEQGFTTLAGGKVVMPPIMTLGVPDHNGEVCVKTAYVPGIDSFAM
KMSPGFFDNPKIGLPSTTGLMVVFSSRTGILEALLLDNGYLTDVRTAAAGAVAARHLARAEASHVCIIGA

GVQARMQLQAMTLVRDIESAAIWARDVAKAEAAAASLRQDLGIDVTVGTDIAQAVSPADIVITTTPASSP
VIRAEWLQPGQLVIAMGSDQEHKGELEPACLTKADLYVPDSQAQCALKGELRSAIEAGLIAAGQSFAELG
QVTSGQTPGRRSDRELIIADLTGTGVQDTAIATLAGNRADATGAGATFTS

>WP_011046887.1 ectoine hydrolase DoeA ; eutD [Ruegeria pomeroyi]
MADPKLFFTRAEFQARLDKTRAEMARRGLDLLIVTDPSNMNWLTGYDGWSFYVHQCVVLTLEGEPLWYGR
GQDANGALRTCFMDPVNIIGYPDHYVQSTERHPMDYLSARLKDRGLDGGVIGVEMDNYWFTAAAFMSLQK
HLPNARFTDATALVNWQRAVKSEAELTYMRQAGKIVERMHQRIFERVEPGMRKCDLVADIYDAGLRYDAG
LGFGGDYPAIVPLLPSGSDAAAPHLTWDDLPMKSGEGTFFEIAGVMHRYHCPLSRTVFLGKPTQTFLDAE
KAVLEGMEAGLEMARAGNTCEDIAKAFFKVLKSYGIEKDSRTGYPIGVSYPPDWGERTMSLRPGDTTVLQ
ENMTFHFMTGLWMEDWGFEITESIRIGADGPECLSNVPRKMFVKD

>WP_011046886.1 N(2)-acetyl-L-2,4-diaminobutanoate deacetylase DoeB ;
EutE [Ruegeria pomeroyi]
MQKNPISPTIPLDRDGVFHGFLKLPHSRDDSAWGSVMIPLTVIKNGAGPTALLTGANHGDEYEGPVALHE
LAATTSAEDVTGRLIIVPAFNYPAFRAGSRTSPIDRGNLNRSFPGRPDGTVTEKIADYFQRTLLPMADLA
VDFHSGGKTLDFVPFAAAHILEDKATQAACFAAMKAFNAPYSVELLEIDSAGMYDTAVEEMGKVLVTTEL
GGGGSSSARSNAIAKKGLRNVLIHAGILKGEMQLDETVNLTMPDDDCFVFSEGDGLFEMMIDLGAPVAKG
DLLARVWPLDRTGQPPVEYRARRAGLVISRHFPGLIKSGDCVAVVGVTGA

>WP_011046885.1 Lrp/AsnC family transcriptional regulator ; AsnC
[Ruegeria pomeroyi]
MQLDQRDLDILRVLSTEGRITKAALADRIGLSPTPCWDRLKKLEQAGLIEGYGARINLKKLAPHVTVFVA
AEIADHTAASFRAFEAAMQRYEEVTACWALGGGFDYLLQIVTRDIDAYQRLIDEMLDDRIGLSRYFTYVV
TKPVKGTGAPPLKILLGLE

>WP_011046884.1 NAD-dependent succinate-semialdehyde dehydrogenase ;
ssd [Ruegeria pomeroyi]
MTETALSARADIADKALVRSFSYINGKWCAAANGETFAVTDPADGTELGHAASLSAEESSAAVDAAQAAF
PAWAGRLPQERAALLRRWYELLLEHKEDLARIMVLEQGKPLSEARGEIDYGAAFVEFYAEEAKRPNIEGV
TSHLPDAEVELWREPVGVAALITPWNFPSAMLTRKAAAALAAGCTVVAHPSRETPFSALALAELAERAGV
PPGVFNVVTGTAATVVEPWTRDTRVRALSFTGSTEIGKLLYRQSADTVKRLVMELGGHAPVIVFKDCDLD
KAVSETIKAKFATSGQDCLGANRILVERPVYADFCARFTLAAQALTLGPGMADCDLGPLMNEQAVQKQED
HVADALARGARLACGGRRHPRGPLFYEPTVLVDVPPDALIMSQETFGPVAAIAPFDTEEEAVARANDSEY
GLVAYVHSNDPRRIYRLSRALQYGMVAVNRTKVTGAPIPFGGTKQSGLGREGARLGMEAFTEVKYICRDW
A

>WP_011046883.1 aspartate aminotransferase family protein ; atf
Ruegeria pomeroyi]
MLKNDQLDQWDRENFFHPSTHLAQHARGDSANRVIKTASGVFIEDRDGNKLLDAFAGLYCVNVGYGRQEI
ADAIADQARELAYYHSYVGHGTEASITLAKMILDRAPANMSKVYFGLGGSDANETNVKLIWYYNNILGRP
EKKKIISRWRGYHGSGLVTGSLTGLELFHKKFDLPVNQVIHTEAPYYFRRADPDQSEAQFVAHCAAELEA
LIEREGADTIAAFIGEPVLGTGGIVPPPAGYWEAIQAVLRKHDILLIADEVVTGFGRLGTMFGSDHYGIE
ADIITIAKGLTSAYAPLSGSIISDKVWKVLEQGTDENGPIGHGWTYSAHPIGAAAGVANLKLIDRLNLVQ
NAGETGAYLNATMTEALAGHPNVGEVRGAGMLCAVEFVKDKDSRLFFDAADKIGPQISAKLLEQDKVIAR
AMPQGDILGFAPPFCLSRAEADQVVDATLRAVRTVLG

>ABX39523.1 diaminobutyric acid acetyltransferase ; EctA [Halobacillus
halophilus DSM 2266]
MISIPTTTKEVKKYQFCEPEKSDGAEVYEMIENIPILDLNSSYSYLLWCEFFSDTSVVVKDEGNTVGFIS

GFIHPSSPDTLFIWQVAVAESERGQGLATRMIDHIVGRHEDVNYIEATVSPSNKPSQQLFKGFAKKKEVP
CDISVCFEEDDFPGDDHEQENTFKIGPLKN

>ABX39524.1 diaminobutyrate-2-oxoglutarate transaminase ; EctB [Halobacillus halophilus DSM 2266]
MTNSNQKTDMSVFENLESEVRSYSRSFPTTFEKAKGYHMWDGNGNSYIDFFAGAGALNYGHNDDAMQQKL
IEYIQSDGLLHSLDMATNARAEFLKKFNEVILEPRNMSYKIMFPGPTGTNTVESALKIARKVTGREKVVT
FTNAFHGMTIGALSVTGNAFKRHGAGIPLTYSMAMPYDDYMDGYDSLDYLERMIQDGGSGIDLPAAIILE
TVQGEGGINAASFEWLKKVEELCRRLDIMLIVDDVQAGCGRTGTFFSFEKAGISPDVVCLSKSISGSGLP
MAITLIKPEYDQWGPGEHNGTFRGNNLAFVTATEALDYWKTNEFSESIEEKSGLFQKRLYQFVKDYPELE
GEVRGRGLMLGIASKKEGLAGQIAAESFKKGLIIETSGPDDEVLKMLPPLIIDEEGISKGLDIIEESIKA
VLGKEEAKV

>ABX39525.1 ectoine synthase ; EctC [Halobacillus halophilus DSM 2266]
MNVVKLEDLIGTEREVKDDNWKSRRFLLKDDNVGFSLHDTVLYAGTETYIWYKHHIEAVYCIEGEAEIET
LKDGKKYQIKPGTMYCLDGHEKHYLRAKTDFRVVCVFNPALVGNEVHDEEGVYRLPEESK
