# Supplementary material for: Metagenomic Analysis of a Concrete Bridge Reveals a Microbial Community Dominated by Halophilic Bacteria and Archaea
Source: Microbiol Spectr. 2023 Jul 5;11(4):e05112-22. doi: 10.1128/spectrum.05112-22 (PMC10434110; doi:10.1128/spectrum.05112-22)
Supplement: Supplemental file 6 — Figure S1. Download spectrum.05112-22-s0001.pdf, PDF file, 0.2 MB [file spectrum.05112-22-s0001.pdf]

A)

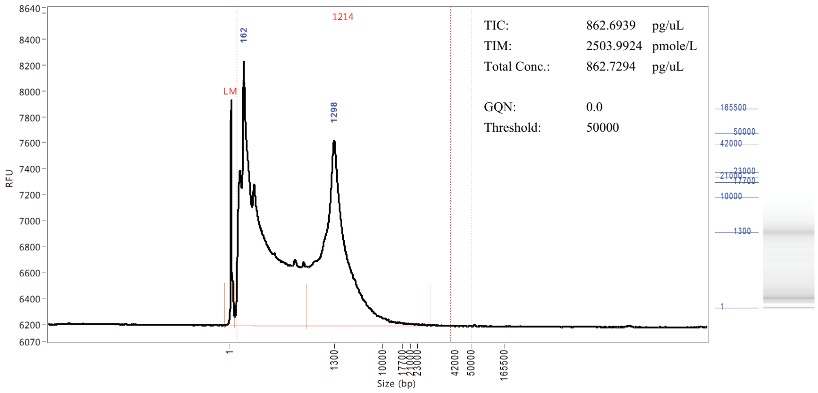

Peak	Size (bp)	Conc. (pg/uL)	From (bp)	To (bp)	Avg. Size (bp)	CV%	RFU	Corr. Peak Area
1	1 (LM)	1.5339	0	50	5	304.91	1738	13.163
2	162	551.1876	50	953	393	62.55	2037	236.496
3	1298	311.5063	953	30065	2610	106.74	1426	133.657
Smear Analysis	76 bp to 40000 bp	857.8519 pg/ul	99.4 %Total	1162.6798 pmole/L	1214 Avg. Size (b.p.)	174.80 %CV		

B)

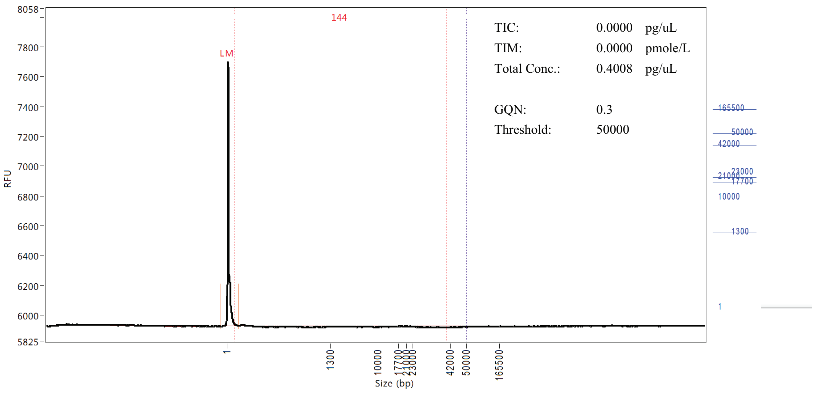

Peak	Size (bp)	Conc. (pg/uL)	From (bp)	To (bp)	Avg. Size (bp)	CV%	RFU	Corr. Peak Area
1	1 (LM)	1.5339	0	146	8	234.37	1775	13.744
Smear Analysis	76 bp to 40000 bp	0.8152 pg/ul	203.4 %Total	9.2973 pmole/L	144 Avg. Size (b.p.)	36.51 %CV		

Figure S1. Extracted DNA analyzed by capillary pulsed-field electrophoresis. DNA extracted from the concrete (A) and negative control (B) samples was profiled by capillary pulsed-field electrophoresis with an Agilent Femto Pulse system prior to library preparation. The fragment length distribution shows that the DNA recovered from concrete was highly fragmented and that negative control DNA was nearly undetectable. Concentration estimates obtained with the same method are below the assay's lower limit of 5 pg uL^{-1} and are possibly due to signal noise, further confirming nearly undetectable negative control yields. The lower marker (LM) peak is from a spiked-in marker required for interpreting the Femto Pulse results.
