# Supplementary material for: Metagenomic Analysis of a Concrete Bridge Reveals a Microbial Community Dominated by Halophilic Bacteria and Archaea
Source: Microbiol Spectr. 2023 Jul 5;11(4):e05112-22. doi: 10.1128/spectrum.05112-22 (PMC10434110; doi:10.1128/spectrum.05112-22)
Supplement: Supplemental file 7 — Figure S2. Download spectrum.05112-22-s0002.pdf, PDF file, 0.4 MB [file spectrum.05112-22-s0002.pdf]

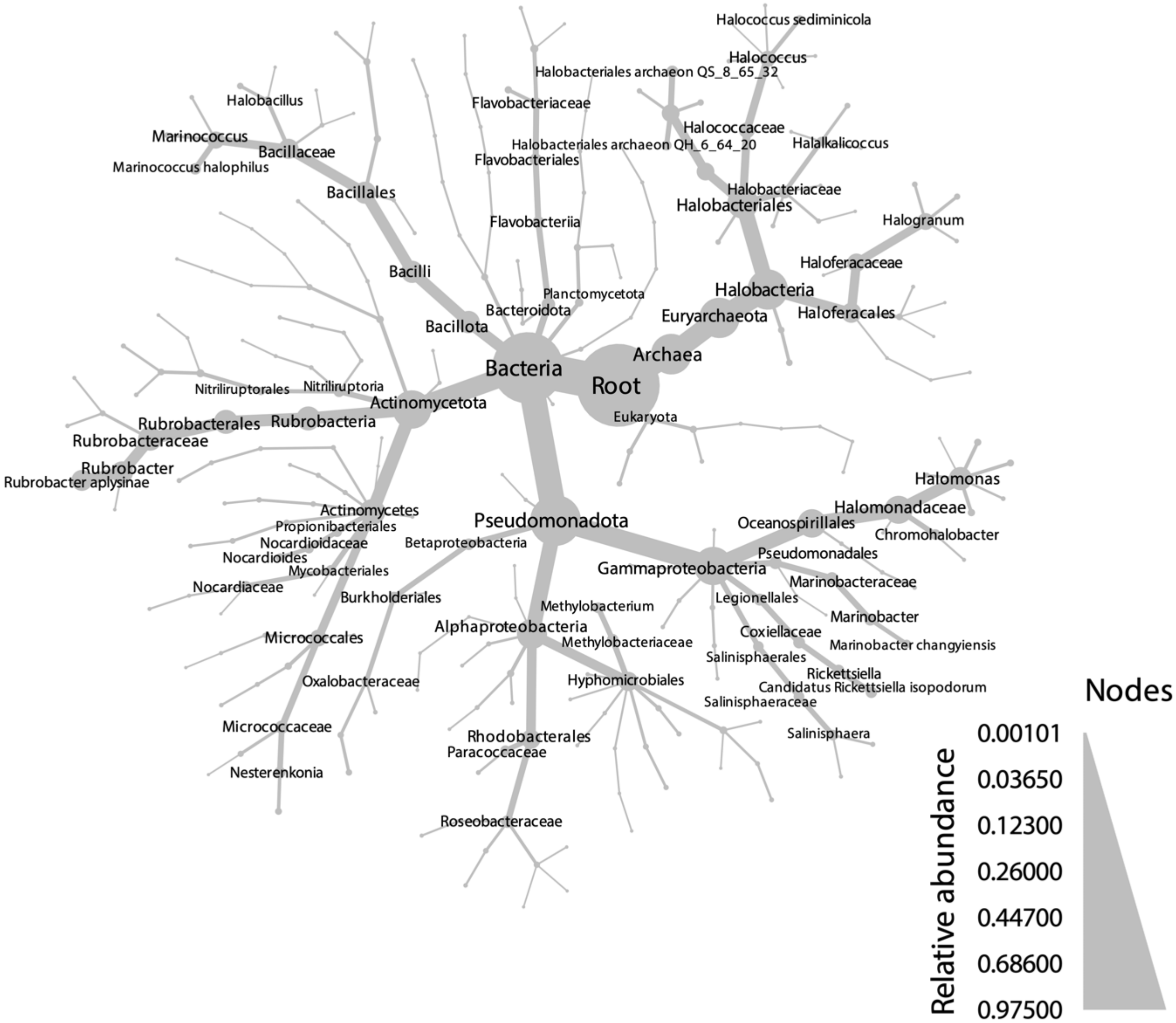

Figure S2. Microbial community composition of concrete determined by read mapping to the UniRef100 protein database.

Bacteria and Archaea were the most abundant organisms while eukaryotes and viruses were observed at much lower abundances, similar to the community profile determined with Kraken2 (Figure 2). Only groups with relative abundances > 0.001 are shown; however, excluded taxa were included in abundance calculations of higher-rank taxa. While the Kraken2 estimates may offer more specific estimates due to the use of genome reference databases, the UniRef100 database is more exhaustive.
