# Supplementary material for: Metagenomic Analysis of a Concrete Bridge Reveals a Microbial Community Dominated by Halophilic Bacteria and Archaea
Source: Microbiol Spectr. 2023 Jul 5;11(4):e05112-22. doi: 10.1128/spectrum.05112-22 (PMC10434110; doi:10.1128/spectrum.05112-22)
Supplement: Supplemental file 8 — Figure S3. Download spectrum.05112-22-s0003.pdf, PDF file, 2.1 MB [file spectrum.05112-22-s0003.pdf]

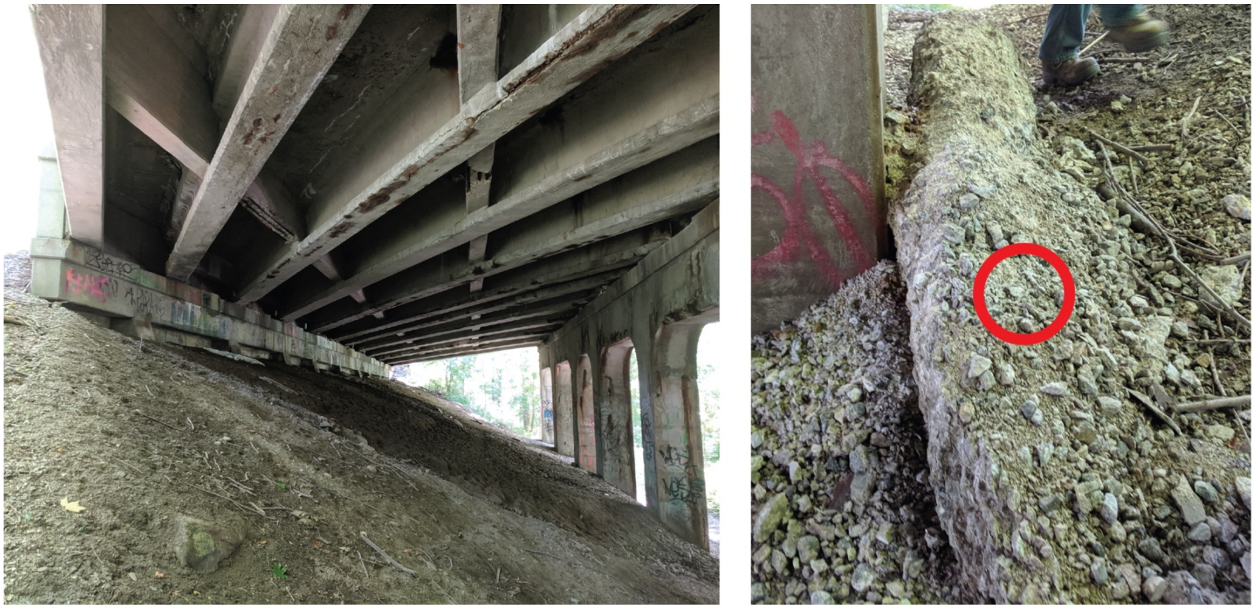

Figure S3. Sample site.
The concrete sample analyzed was collected from a New Jersey road bridge (left). Material was collected just out of frame to the right of the left image from a piece of concrete that had fallen from underside of the bridge deck (right; approximate sampled area is circled). The top layer of material was removed prior to sampling.
